# Diagnostic Immunohistochemistry of Soft Tissue and Bone Tumors: An Update on Biomarkers That Correlate with Molecular Alterations

**DOI:** 10.3390/diagnostics11040690

**Published:** 2021-04-12

**Authors:** William J. Anderson, Vickie Y. Jo

**Affiliations:** Department of Pathology, Brigham and Women’s Hospital and Harvard Medical School, 75 Francis Street, Boston, MA 02115, USA; WANDERSON@BWH.HARVARD.EDU

**Keywords:** immunohistochemistry, sarcoma, soft tissue tumors, bone tumors, translocation, mutation, gene rearrangement, fusion gene

## Abstract

The diagnosis of benign and malignant soft tissue and bone neoplasms is a challenging area of surgical pathology, due to the large number, rarity, and histologic diversity of tumor types. In recent years, diagnosis and classification has been aided substantially by our growing understanding of recurrent molecular alterations in these neoplasms. Concurrently, the role of diagnostic immunohistochemistry has also expanded, with the development of numerous biomarkers based on underlying molecular events. Such biomarkers allow us to infer the presence of these events and can therefore substitute for other ancillary molecular genetic techniques (e.g., fluorescence in situ hybridization, polymerase chain reaction, and next-generation sequencing). In this review, we discuss a range of biomarkers currently available for these neoplasms, highlighting the accuracy, staining characteristics, and interpretation pitfalls of each antibody. These include immunohistochemical antibodies that represent reliable surrogates for the detection of gene fusions (e.g., STAT6, CAMTA1, FOSB, DDIT3) and more recently described breakpoint-specific antibodies (e.g., SS18-SSX, PAX3/7-FOXO1). Additionally, discussed are markers that correlate with the presence of gene amplifications (e.g., MDM2, CDK4), deletions (e.g., SMARCB1, SMARCA4), single nucleotide variants (e.g., G34W, K36M), aberrant methylation (H3K27me3), and increased expression as discovered through gene expression profiling (e.g., MUC4, DOG1, ETV4, NKX2.2, NKX3.1).

## 1. Introduction

Sarcomas are a large and heterogeneous group of malignant mesenchymal neoplasms, comprising at least 70 distinct types, which together account for around 1% of all cancers [1]. The diagnosis of sarcomas can be challenging, due in part to the rarity of each tumor type, their remarkable histologic diversity, and the frequent use of limited biopsy material. Despite these challenges, correct classification remains crucially important for patient management, prognostication, and research efforts. 

In the last three decades, a broad repertoire of molecular events has been characterized in sarcomas. Many of these are found recurrently and consistently in certain tumor types, allowing ancillary genetic tests to become very helpful in the diagnosis of difficult cases. These include karyotyping, fluorescence in situ hybridization (FISH), reverse transcriptase-polymerase chain reaction (RT-PCR), and more recently, next-generation sequencing-based platforms. Concurrently, immunohistochemistry—a more rapid, relatively inexpensive, and widely available technique—has continued to evolve and remain at the forefront of sarcoma diagnostics. In recent years, this has largely been due to a wave of new biomarkers, able to detect the protein products of specific genetic alterations and thereby clearly exploit our growing understanding of molecular pathology. For many sarcomas, this approach is affording more precise immunohistochemical diagnosis than was possible with earlier markers which generally aim to establish line of differentiation. Many markers now act as convenient surrogates for molecular techniques by allowing the detection of gene fusions (the largest category of recurrent alterations in sarcomas), as well as amplifications, deletions, single nucleotide variants (SNV) and aberrant methylation (Table 1). In this review, we discuss the novel and more established surrogate markers currently available in each of these molecular categories, highlighting their accuracy, staining characteristics, and interpretation pitfalls.

## 2. Surrogate Markers of Gene Fusions

### 2.1. CCNB3 in BCOR-Rearranged Sarcoma

In the recent WHO classification (2020), a new category for ‘undifferentiated small round cell sarcomas of bone and soft tissue’ was introduced [1]. This incorporated Ewing sarcoma (ES) alongside more recently characterized tumors that show overlapping morphology with ES (hence their prior designation as ‘Ewing-like’) and distinct molecular features. These include sarcomas with *BCOR* alterations, *CIC*-rearranged sarcoma (see ‘WT1 and ETV4’), and round cell sarcoma with *EWSR1*-non-ETS fusions. Round cell sarcoma with *BCOR-CCNB3* fusion, first described in 2012 [2], is most common in children and has a strong predilection for males. Histologically, it consists of small round to ovoid (and occasionally spindled) cells with a diffuse growth pattern and a prominent vascular network [3]. Immunohistochemistry for CCNB3 (cyclin B3) was shown to have excellent sensitivity (100%) and specificity (100%) in the original series, with all fusion-positive cases showing strong and diffuse nuclear positivity [2]. However, in a follow-up study, CCNB3 expression was reported to be seen in rare subsets of some other spindle and round cell tumors, including solitary fibrous tumor (15%), ES (1/18 cases), rhabdomyosarcoma (1/12 cases), and (adult-type) fibrosarcoma (1/11 cases) [4]. 

### 2.2. BCOR in BCOR-Rearranged Sarcoma

Immunohistochemistry for BCOR (BCL6 corepressor) has also been developed and is very sensitive for round cell sarcomas with *BCOR-CCNB3* [4,5]. In addition, it has the advantage of being positive in the wider family of tumors which share BCOR overexpression due to *BCOR* internal tandem duplications (ITD) or other gene fusions; examples include clear cell sarcoma of kidney and primitive myxoid mesenchymal tumor of infancy. BCOR expression is quite rare in other tumor types, apart from synovial sarcoma (49%) [5]. Of note, the group of tumors with BCOR upregulation are also commonly positive for SATB2 [5] (discussed later), cyclin D1 and TLE1.

### 2.3. WT1 (C-Terminus) in Desmoplastic Small Round Cell Tumor

**Figure 1:**
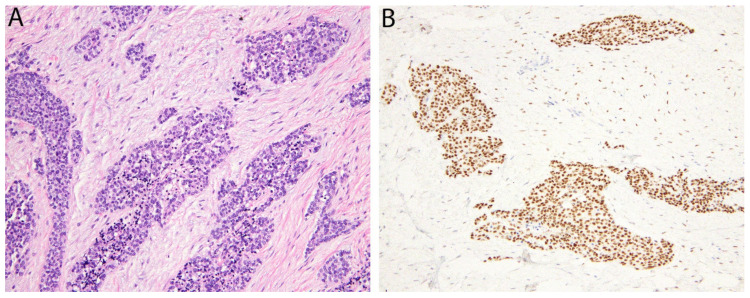
Desmoplastic small round cell tumor. (**A**) Irregular islands of monotonous tumor cells with small round nuclei are separated by a hypocellular desmoplastic stroma. (**B**) The tumor cells show nuclear positivity for the WT1 C-terminus.

Desmoplastic small round cell tumor (DSRCT) is an aggressive sarcoma with a predilection for children and young adults, particularly males, and which often presents as an abdominal cavity mass [6]. Microscopically, DSRCT has tumor cells with round nuclei arranged in islands or nests that are separated by prominent bands of fibrous/desmoplastic stroma (Figure 1). Shortly after its initial description in 1991, a recurrent t(11;22)(p13;q12) translocation was identified [7], which leads to an *EWSR1-WT1* gene fusion in more than 95% of cases. Since the encoded fusion protein includes the C-terminus of the Wilms’ tumor protein (WT1), antibodies against this region (as opposed to the more commonly used clones directed against the N-terminus) have become very useful in supporting the diagnosis, as almost all cases show strong nuclear expression [8]. 

### 2.4. PAX3/7-FOXO1 in Alveolar Rhabdomyosarcoma

Another round cell sarcoma, alveolar rhabdomyosarcoma (ARMS), exhibits skeletal muscle differentiation and is frequently driven by *FOXO1* gene fusions [9]. These fuse *FOXO1* with either *PAX3* or *PAX7*, transcription factors which both have roles in normal myogenesis [10]. ARMS occurs most often in the extremities of adolescents and young adults, but can also be encountered in the head and neck and paraspinal regions. Histologically, the tumor cells are typically arranged in nests with central areas of discohesion which impart an ‘alveolar’ appearance. In challenging cases, *FOXO1* FISH using break-apart probes has traditionally been utilized to aid diagnosis. Very recently however, immunohistochemistry has been applied for the recognition of PAX3/7-FOXO1 fusions using antibodies directed against an epitope at the junction of PAX3/7 and FOXO1, thought to be unique to the fusion protein [11]. One of the two clones generated (PFM.2) demonstrated 91% sensitivity and 100% specificity. Interestingly, while diffuse positivity was observed in cases with *PAX3-FOXO1*, more focal staining was seen in those with *PAX7-FOXO1*. Previously, cross-reactivity for PAX5 and PAX3 antibodies has been reported, although these were not widely adopted in diagnostic practice [12,13]. 

### 2.5. PAX3 in Biphenotypic Sinonasal Sarcoma

Biphenotypic sinonasal sarcoma (BSNS) is a recently recognized low-grade sarcoma of the sinonasal tract that demonstrates neural and myogenic differentiation by traditional immunohistochemistry (S100 and SMA positivity) [14]. Histologically, it usually consists of a monomorphic population of spindle cells arranged in intersecting fascicles that may infiltrate normal sinonasal epithelium and bone. Recurrent *PAX3* gene fusions, the most common being *PAX3-MAML3*, have been identified in the vast majority of BSNS, helping to define it as a distinct entity [15,16]. Immunohistochemistry for PAX3, using a monoclonal antibody, was recently shown to be highly sensitive and specific for BSNS and is a useful substitute for *PAX3* break-apart FISH [17].

### 2.6. STAT6 in Solitary Fibrous Tumor

Solitary fibrous tumor (SFT) is a fibroblastic neoplasm of variable biologic potential that can arise at a wide range of anatomic sites. Histologically, it is typically composed of spindled-to-ovoid cells with a haphazard growth pattern and a prominent network of ectatic (‘staghorn’) blood vessels. In 2013, two groups discovered that SFT is genetically defined by a *NAB2-STAT6* gene fusion [18,19]. Despite this major advance, the molecular diagnosis of SFT did not become straightforward, since *NAB2-STAT6* is generated by a small paracentric inversion of 12q13 that cannot be detected by FISH, while the variable breakpoints also make RT-PCR laborious. Fortunately, soon after the fusion was characterized, several studies established the utility of STAT6 immunohistochemistry as a surrogate marker [20,21,22]. It has become widely adopted as a very sensitive and specific means of supporting the diagnosis. Of note, a small subset of dedifferentiated liposarcomas (DDLPS) may show expression since the *STAT6* gene may be co-amplified with its neighboring genes, *MDM2* and *CDK4* [23].

### 2.7. ALK in Inflammatory Myofibroblastic Tumor 

**Figure 2:**
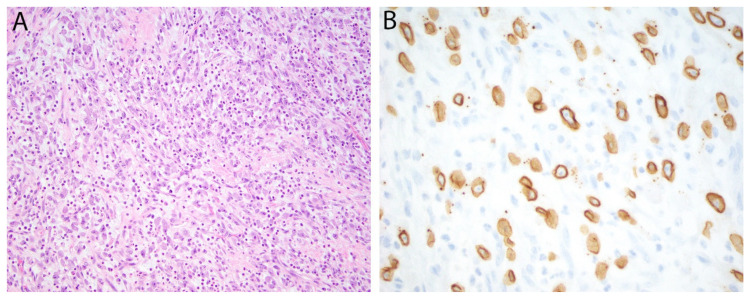
Epithelioid inflammatory myofibroblastic sarcoma. (**A**) Epithelioid tumor cells with vesicular nuclei and prominent nucleoli are admixed with numerous neutrophils. (**B**) Immunohistochemistry for ALK is positive in a nuclear membrane distribution consistent with an underlying *RANBP2-ALK* gene fusion.

The anaplastic lymphoma kinase gene (*ALK*), encoding ALK, is located on 2p23 and is rearranged in around 50–60% of inflammatory myofibroblastic tumors (IMT) [24]. IMT is a neoplasm of intermediate (rarely metastasizing) malignancy composed of fibroblastic/myofibroblastic spindle cells admixed with an inflammatory cell infiltrate. *ALK* rearrangements in IMT lead to gene fusions with a long list of potential 5′ partners. These have in common the ability to homodimerize, bringing the ALK portions of the fusion protein together and activating downstream signaling. Immunohistochemistry for ALK is positive in the majority of cases. Cytoplasmic staining is most common, however, alternative staining patterns can be seen in association with specific underlying gene fusions, such as granular cytoplasmic (*CLTC-ALK*), nuclear membrane (*RANBP2-ALK*) or perinuclear (*RRBP1-ALK*). The latter two are characteristically seen in an aggressive IMT variant termed epithelioid inflammatory myofibroblastic sarcoma (Figure 2) [25]. 

Immunohistochemistry for ALK is also useful in the diagnosis of other mesenchymal lesions known to harbor recurrent *ALK* rearrangement. These include epithelioid fibrous histiocytoma (EFH) [26], non-neural granular cell tumor [27], pseudosarcomatous myofibroblastic proliferations of the bladder [28], ALK-positive histiocytosis [29], and a subset of gastrointestinal leiomyomas [30]. A small subset of IMT that are negative for *ALK* rearrangement harbor alternative *ROS1* fusions (as can occur in lung adenocarcinomas) and are positive for ROS1 immunohistochemistry. As in lung adenocarcinomas harboring *ALK* or *ROS1* rearrangement, targeted therapy with Crizotinib has been associated with objective responses in IMT [31,32].

### 2.8. Pan-Trk in Infantile Fibrosarcoma and NTRK-Rearranged Spindle Cell Neoplasms 

Gene fusions involving *NTRK1*, *NTRK2* or *NTRK3*—which encode the TrkA, TrkB and TrkC receptor kinases, respectively—are found throughout a wide range of human neoplasms. Among mesenchymal tumors, they are seen in infantile fibrosarcoma (IFS) as well as a separate, emerging category of *NTRK*-rearranged spindle cell neoplasms [1]. IFS is a fibroblastic/myofibroblastic neoplasm of intermediate biologic potential that arises in neonates and infants and characteristically harbors an *ETV6-NTRK3* gene fusion [33]. Microscopically, it typically consists of intersecting highly cellular fascicles of spindle cells arranged in a ‘herringbone’ pattern. On the other hand, the distinct group of *NTRK*-rearranged spindle cell neoplasms has a broader morphologic spectrum. A subset resembles lipofibromatosis (lipofibromatosis-like neural tumor) and shows diffuse infiltration of subcutaneous adipose tissue, while others have a more solid growth pattern of uniform spindle cells with characteristic hyalinization of perivascular and stromal collagen. Co-expression of S100 and CD34 is often a further diagnostic clue. Recently, immunohistochemistry using a monoclonal antibody directed against the three Trk proteins (‘Pan-TRK’), has been introduced as a potential surrogate for genetic confirmation [34]. However, although tumors with underlying *NTRK* rearrangement often exhibit cytoplasmic and/or nuclear positivity for Pan-Trk, similar staining can be seen in morphologic mimics [35]. Therefore, its low specificity generally means that molecular testing is still required to confirm the diagnosis. Clinically, certain patients with IFS or other sarcomas with *NTRK* rearrangement may now benefit from targeted therapy with the TRK inhibitor Larotrectinib, making the accurate diagnosis of these neoplasms of further importance [36,37].

### 2.9. SS18-SSX/SSX C-Terminus in Synovial Sarcoma

**Figure 3:**
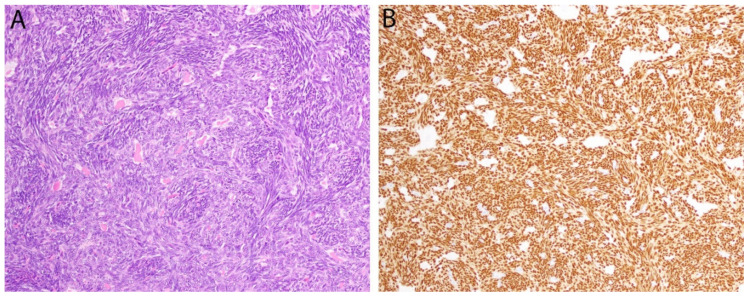
Synovial sarcoma. (**A**) In this biphasic tumor, a population of monomorphic spindle cells is present along with occasional gland-like spaces containing eosinophilic material. (**B**) Diffuse positivity for the breakpoint-specific antibody SS18-SSX is observed in both components.

Synovial sarcoma is a spindle cell sarcoma that arises most commonly in the deep soft tissue of adolescents and young adults, often as a circumscribed mass adjacent to a joint (although it is unrelated to synovium). Histologically, it is either composed purely of monomorphic spindle cells (monophasic form), a combination of spindle cells and glandular structures (biphasic form), or predominantly round cells (poorly differentiated). While epithelial differentiation is most apparent in biphasic cases (Figure 3), it can also be demonstrated by immunohistochemical expression of epithelial membrane antigen and (less commonly) keratins, regardless of subtype. Synovial sarcoma harbors a t(X;18)(p11;q11) translocation that produces gene fusions between *SS18* and *SSX1, SSX2* or *SSX4*, that have not been identified in other neoplasms. Recently, a fusion-specific antibody (clone E9X9V) that recognizes the junction of the SS18-SSX fusion proteins has been shown to be highly sensitive (95%) and specific (100%) for synovial sarcoma, which shows strong and diffuse nuclear positivity (Figure 3). In the same study, a second antibody against a conserved C-terminus region of the proteins SSX1, SSX2 and SSX4 (clone E5A2C), which represent the 3 fusion partners, showed 100% sensitivity. These results have since been corroborated by other groups and it now seems that the combination of SS18-SSX and SSX C-terminus immunohistochemistry even has the potential to replace molecular testing [38,39].

### 2.10. CAMTA1 in Epithelioid Hemangioendothelioma

**Figure 4:**
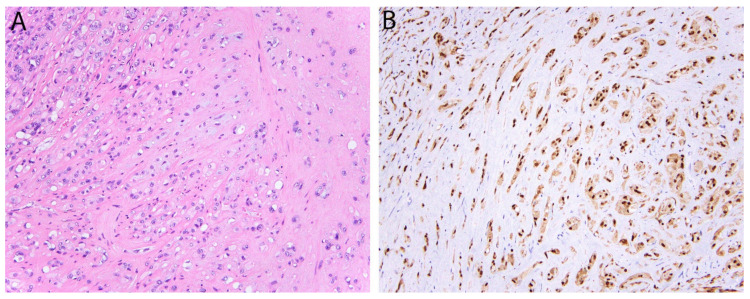
Epithelioid hemangioendothelioma with *WWTR1-CAMTA1* fusion. (**A**) Tumor cells are arranged in cords and single cells in a myxohyaline stroma. Occasional intra-cytoplasmic vacuoles are also present. (**B**) Strong and diffuse nuclear expression of CAMTA1.

Epithelioid hemangioendothelioma (EHE) is a malignant vascular neoplasm that arises in bone, soft tissue and viscera (particularly the lungs and liver) [40]. Microscopically, it typically consists of epithelioid cells arranged as cords and single cells in a myxohyaline stroma, while blood vessel formation is not evident (Figure 4). The majority (more than 90%) harbor a recurrent t(1;3)(p36.23;q25.1) translocation resulting in a *WWTR1-CAMTA1* gene fusion [41]. Nuclear expression of calmodulin-binding transcription activator 1 (CAMTA1) is seen in almost all cases and has become a dependable means to support the diagnosis [42]. Of note, CAMTA1 is negative in a small subset of EHE with a different gene fusion (see below, Section 2.11) [43].

### 2.11. TFE3 in Alveolar Soft Part Sarcoma, PEComa, and Epithelioid Hemangioendothelioma

Transcription Factor E3 (TFE3) is a member of the MiT/TFE family of transcription factors and is involved in the pathogenesis of several sarcoma subtypes through gene rearrangement. *TFE3* fusions were originally identified in alveolar soft part sarcoma (ASPS) [44], in the form of *ASPSCR1-TFE3,* which is generated by a t(X;17) translocation [44]. ASPS is a high-grade sarcoma that typically arises in the deep soft tissues of the extremities, trunk, or head and neck region. Microscopically, it comprises epithelioid tumor cells with eosinophilic cytoplasm arranged in a strikingly nested or organoid pattern. Nuclear positivity for TFE3 is seen due to the presence of the fusion protein.

**Figure 5:**
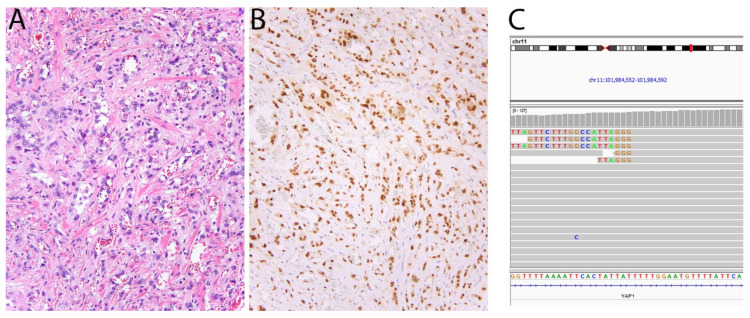
Epithelioid hemangioendothelioma with *YAP1-TFE3* fusion. (**A**) In contrast with conventional EHE, this tumor shows blood vessel formation and cells with abundant eosinophilic cytoplasm. (**B**) The tumor cells show strong nuclear expression of TFE3. (**C**) Screenshot of *YAP1* in Integrative Genomics Viewer showing several reads mapping to *TFE3*.

More recently, a variant of EHE, accounting for less than 5% of cases, has also been recognized to harbor *TFE3* fusions (*YAP1-TFE3*) [43]. Histologically, it differs from conventional EHE in that it often comprises epithelioid tumor cells with voluminous eosinophilic cytoplasm that line well-formed vascular spaces (Figure 5). In this variant, immunohistochemistry for TFE3 is also a relatively sensitive marker [43]. At times however, the intensity of staining can be weak and challenging to interpret, while it is also hampered by imperfect specificity. Finally, rearrangement of *TFE3* is also characteristic of a subset of PEComas [45], for which TFE3 immunohistochemistry can also be used.

### 2.12. FOSB/FOS in Pseudomyogenic Hemangioendothelioma

**Figure 6:**
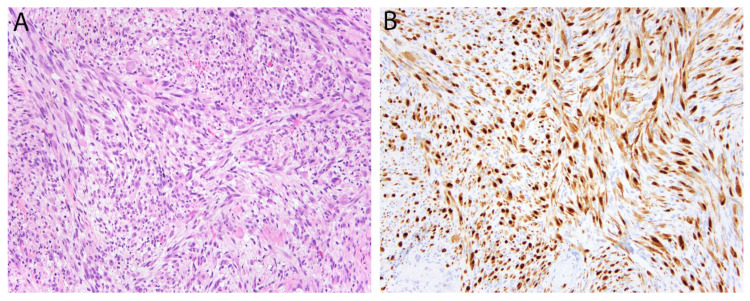
Pseudomyogenic hemangioendothelioma. (**A**) The tumor is composed of loose intersecting fascicles of spindle cells with eosinophilic cytoplasm imparting a myoid appearance. (**B**) The tumor cells show diffuse nuclear positivity for FOSB.

Pseudomyogenic hemangioendothelioma (PHE) is a vascular neoplasm of intermediate biologic potential that was initially described in 1992 but fully characterized nearly two decades later [46,47]. It shows a predilection for young adult males, as well as a striking tendency to arise multicentrically in different anatomic compartments (ie. subcutis, deep soft tissue, and bone) of a single limb [47]. In 2014, a recurrent t(7;19)(q22;q13) translocation was identified in most cases of PHE, producing a *SERPINE1-FOSB* gene fusion [48]. More recently, alternative *FOSB* fusions have been described in rarer examples (*ACTB-FOSB*, *CLTC-FOSB,* and *WWTR1-FOSB*) evidencing the oncogenic importance of this gene in PHE [49,50,51]. The FOSB protein (along with the related proteins FOS, FOSL1 and FOSL2) normally combines with JUN to function as a transcription factor complex (AP-1). In PHE, *FOSB* rearrangements lead to increased expression of FOSB such that almost all cases show strong and diffuse nuclear positivity (Figure 6) [52]. In addition, FOSB is a useful biomarker for epithelioid hemangioma, a separate vascular neoplasm that behaves in a benign fashion [53]. 

Another AP-1 transcription factor gene, *FOS,* has recently been shown to be rearranged in osteoid osteoma and osteoblastoma [54]. Immunohistochemistry for FOS can aid in the important distinction of these benign bone tumors from osteosarcoma [55]. 

### 2.13. DDIT3 in Myxoid Liposarcoma

**Figure 7:**
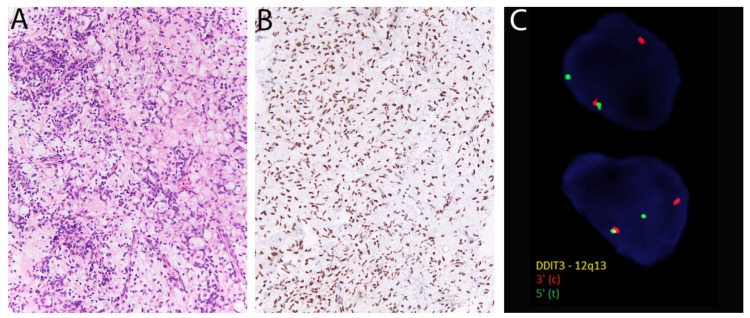
Myxoid liposarcoma. (**A**) There are round-to-ovoid tumor cells, univacuolated lipoblasts, and prominent thin-walled blood vessels. (**B**) Diffuse nuclear positivity for DDIT3. (**C**) *DDIT3* rearrangement is confirmed with fluorescent in situ hybridization using break-apart probes (Courtesy of Dr. Paola Dal Cin).

Myxoid liposarcoma accounts for around 25% of liposarcomas and is defined by recurrent *FUS-DDIT3* gene fusion. Histologically, it consists of spindled-to-ovoid tumor cells with uniform nuclei distributed in a prominent myxoid matrix alongside thin-branching, or so-called “crow’s feet”, blood vessels (Figure 7). High-grade myxoid liposarcoma demonstrates areas of increased cellularity and round cell morphology (previously known as ‘round cell liposarcoma’) and can be more challenging to recognize. In these cases in particular, molecular confirmation with FISH (using *DDIT3* or *FUS* break-apart probes), may be necessary to distinguish it from morphologic mimics. Very recently, immunohistochemistry for DDIT3 (DNA Damage-Inducible Transcript 3) has emerged as a useful surrogate for molecular testing. Scapa et al. reported DDIT3 positivity in 46/46 cases of myxoid liposarcoma and only limited weak expression in other tumors [56]. Minimal staining was observed in cases of dedifferentiated liposarcoma (15%) and solitary fibrous tumor (25%), possibly due to the proximity of *DDIT3*, *MDM2* and *STAT6* on chromosome 12. Of note, since the comparison group consisted mostly of tumors with myxoid stroma and/or adipocytic differentiation, rather than tumors with round cell morphology, further studies may be warranted to confirm the specificity of DDIT3 expression in mimics of high-grade myxoid liposarcoma.

## 3. Immunohistochemical Markers of Gene Amplification or Inactivation

### 3.1. MDM2 and CDK4 in Atypical Lipomatous Tumor/Well Differentiated Liposarcoma and Dedifferentiated Liposarcoma

**Figure 8:**
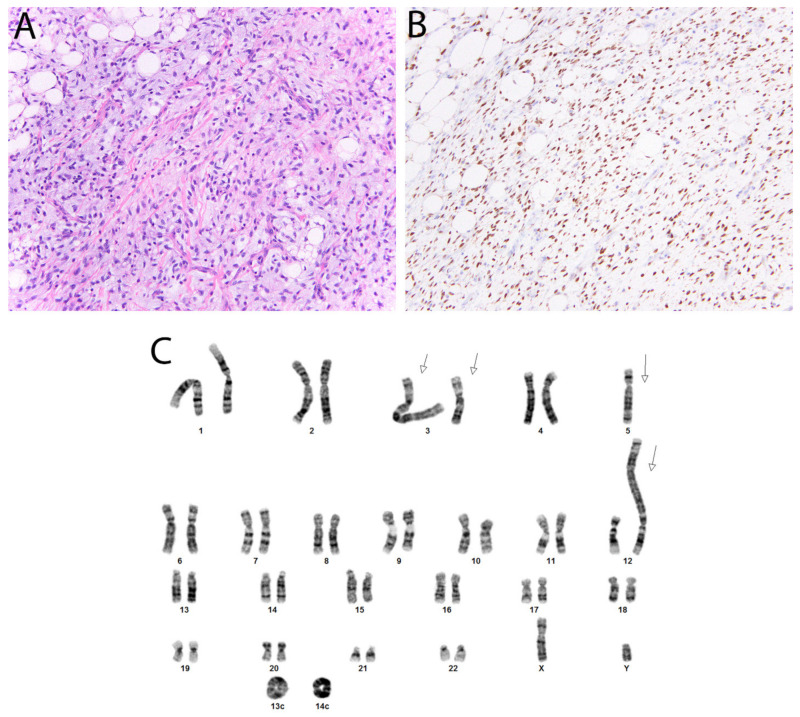
Dedifferentiated liposarcoma. (**A**) In this tumor, there are well-differentiated foci transitioning to a non-lipogenic spindle cell neoplasm. (**B**) Nuclear staining with CDK4 and MDM2 (not shown) is typically seen. (**C**) Karyotype, sec demonstrating several giant marker chromosomes (arrows) and ring chromosomes (Courtesy of Dr. Paola Dal Cin).

Atypical lipomatous tumor/well differentiated liposarcoma and dedifferentiated liposarcoma are the most common forms of liposarcoma and occur most often in the extremities and retroperitoneum. Genetically, they are characterized by amplifications of a region of chromosome 12 (12q13-15) that are evident as ring and giant marker chromosomes by karyotype (Figure 8). The amplified locus encompasses *MDM2*, *CDK4* and less commonly *HMGA2;* molecular confirmation can therefore also be obtained with FISH for MDM2. As a surrogate, immunohistochemistry for MDM2 (as well as CDK4 and HMGA2) is now commonly used, with most cases showing nuclear positivity. These antibodies are not entirely specific however, since positive staining (albeit usually less extensive) can also be seen in malignant peripheral nerve sheath tumor (MPNST) and myxofibrosarcoma. In addition, background non-neoplastic cells such as histiocytes can stain positively and represent a diagnostic pitfall. Finally, *MDM2* amplification is also a feature of intimal sarcoma and low-grade central/parosteal osteosarcoma, allowing the similar use of MDM2 and CDK4 immunohistochemistry to support their diagnosis.

### 3.2. SWI/SNF: SMARCB1 (INI1) and SMARCA4 (BRG1) in Epithelioid Sarcoma and Others

**Figure 9:**
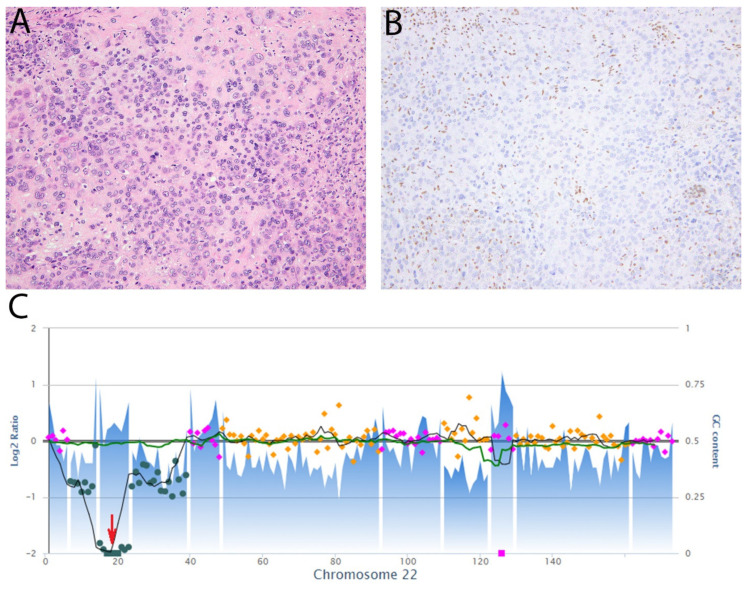
Epithelioid sarcoma, proximal type. (**A**) The tumor comprises epithelioid cells with large vesicular nuclei and prominent nucleoli. Occasional cells have a rhabdoid appearance due to eosinophilic cytoplasmic inclusions. (**B**) Immunohistochemistry for SMARCB1 (INI-1) shows loss of expression while there is retained expression in non-neoplastic cells. (**C**) Copy number plot of chromosome 22 showing two-copy deletion of *SMARCB1* (red arrow).

The switch/sucrose non-fermenting (SWI/SNF) chromatin remodeling complex is a key regulator of gene expression with important tumor suppressive functions, since mutations affecting its various subunits are present in almost a quarter of all cancers [57]. This was first appreciated when recurrent inactivation of SMARCB1 (also known as INI-1 and BAF47, and encoded by *SMARCB1*), were discovered in malignant rhabdoid tumor, an aggressive sarcoma of childhood [58]. Currently, there is a growing list of other mesenchymal tumors in which recurrent *SMARCB1* inactivation has been demonstrated. Chief among these are epithelioid sarcoma (conventional and proximal type; Figure 9) [59], epithelioid MPNST [60], epithelioid schwannoma [60], and poorly differentiated chordoma [61], while it is also a less common feature of extraskeletal myxoid chondrosarcoma [62] and soft tissue myoepithelial tumors [63]. In each of these, immunohistochemistry for SMARCB1 is a reliable surrogate for underlying *SMARCB1* inactivation, since tumor cell nuclei frequently show loss of expression alongside retained expression in non-neoplastic cells. 

SMARCA4 (BRG1) is another SWI/SNF subunit whose encoding gene is recurrently mutated in cancer and for which there is now an immunohistochemical biomarker. Among sarcomas, biallelic *SMARCA4* inactivation is a defining feature of the recently described SMARCA4-deficient undifferentiated uterine sarcoma [64] and sinonasal teratocarcinosarcoma [65]. In addition, SMARCA4 loss of expression can also be observed in tumors more commonly associated with SMARCB1 loss, such as epithelioid sarcoma [66]. Finally, inactivation of *SMARCA4* and subsequent loss of SMARCA4 is also characteristic of rare undifferentiated tumors of the thoracic cavity that were initially described as ‘SMARCA4-deficient thoracic sarcoma,’ though it appears that a significant number of these represent smoking-associated thoracic undifferentiated/de-differentiated carcinomas [67].

### 3.3. SDH in SDH-Deficient Gastrointestinal Stromal Tumor

Succinate dehydrogenase (SDH) is an enzyme complex located in the inner mitochondrial membrane which plays a role in both the tricyclic acid (TCA) cycle and oxidative phosphorylation. It comprises four subunits (SDHA, SDHB, SDHC and SDHD), any of which may be targeted in a subset of gastrointestinal stromal tumors (GIST) leading to SDH deficiency. In general, SDH-deficient GIST is rare, as it essentially only arises in the stomach and represents around 10% of cases at this site, however it accounts for the majority of GIST in the pediatric age group. In contrast with other GIST subtypes, it often has a multinodular/plexiform growth pattern, foci of lymphovascular invasion and associated lymph node metastases. Despite lacking *KIT* and *PDGFRA* mutations, SDH-deficient GIST is positive for both KIT and DOG1 (see below, Section 6.1). Moreover, SDHB immunohistochemistry is now available as a more confident means of identifying tumors with SDH deficiency, since they invariably demonstrate loss of cytoplasmic expression, regardless of which particular subunit is genetically or epigenetically altered. In tumors with loss of SDHB expression, immunohistochemistry for SDHA can also be performed as a second step to further discern those likely to harbor *SDHA* mutation [68]. Overall, most patients with SDH-deficient GIST have germline mutations, some of which may be clinically related to Carney-Stratakis syndrome (the association of GIST and paraganglioma) or Carney triad (GIST, paraganglioma, and pulmonary chondroma). These markers therefore also help to identify patients who may benefit from further assessment by clinical geneticists.

### 3.4. PRKAR1A in Malignant Melanotic Nerve Sheath Tumor and Others 

Malignant melanotic nerve sheath tumor (MMNST), is a rare sarcoma that most often affects adults and arises in association with spinal or autonomic nerves [1,69]. Histologically, it comprises spindled or epithelioid Schwann cells with melanocytic differentiation, hence its former designation as ‘melanotic schwannoma’. The tumor is often heavily pigmented and around 40% show psammomatous calcifications [69]. Genetically, the majority are characterized by germline or somatic inactivation of *PRKAR1A*, a tumor suppressor located on 17q22-24, and a subset are associated with Carney complex (CNC) [70]. Immunohistochemistry for PRKAR1A is diagnostically very useful, particularly with the distinction from melanoma, since MMNST frequently shows loss of expression. It is worth noting that other mesenchymal neoplasms variably associated with CNC may also show loss of PRKAR1A expression, including atrial myxoma and superficial angiomyxoma [71].

## 4. Immunohistochemistry to Identify Epigenetic Alterations

### H3K27me3 in Malignant Peripheral Nerve Sheath Tumor

Malignant peripheral nerve sheath tumor (MPNST) is an aggressive spindle cell sarcoma most commonly occurring in adults, that may be sporadic or arise in association with neurofibromatosis type 1 (NF1) or prior radiation. Recently, MPNST was found to harbor recurrent mutations in *SUZ12* or *EED*, two genes that encode components of the polycomb repressive complex 2 (PRC2) [72]. PRC2 is a key epigenetic regulator responsible for the trimethylation of lysine position 27 on histone 3 (‘H3K27me3′) in normal cells. In MPNST however, the disruption of PRC2 functioning caused by *SUZ12*/*EED* mutation leads to the loss of this trimethylation mark, an event which can now be inferred by immunohistochemistry using antibodies directed against H3K27me3. While normal cells and most other neoplasms show retained nuclear positivity, loss of expression is often seen in MPNST [73,74,75]. However, the overall sensitivity is quite variable, with published values ranging from 36% [74] up to 40% [76]. In general, H3K27me3 loss is more common in tumors of higher grade and in the setting of radiation. Overall, it is very specific for MPNST, but can occasionally be seen in other radiation-associated sarcomas [77] and melanoma [78]. Immunohistochemistry to detect the loss of H3K27 dimethylation (H3K27me2) has been reported to show more discriminatory value between MPNST and mimics [79].

## 5. Immunohistochemical Detection of Single Nucleotide Variants

### 5.1. G34W in Giant Cell Tumor of Bone

**Figure 10:**
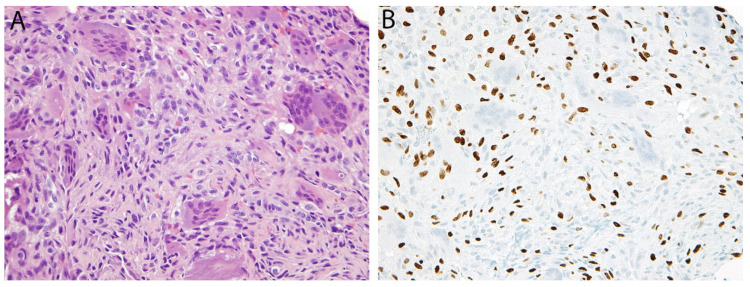
Giant cell tumor of bone. (**A**) Histologically, there is a population of mononuclear cells and scattered osteoclast-type giant cells. (**B**) Immunohistochemistry for G34W is positive in the nuclei of the neoplastic mononuclear cells while the giant cells are negative.

Giant cell tumor of bone (GCTB) is a locally aggressive tumor that characteristically arises in the epiphyses of skeletally mature individuals. In 2013, greater than 90% of GCTB were found to harbor mutations in *H3-3A* (*H3F3A*), affecting the glycine 34 residue of the encoded histone protein [80]. The large majority of these are as a p.Gly34Trp (p.G34W) substitution, which is very rare in other bone tumors [81]. Subsequently, an antibody has been developed to recognize the mutated G34W residue (Figure 10). G34W immunohistochemistry is very sensitive and specific for GCTB, although a subset (<10%) of cases harboring alternative substitutions (e.g., p.G34L, M or V)—often those arising in the small bones of the hands and feet—can be negative [82]. In addition, positivity can be observed in rare cases of GCTB that have undergone malignant transformation. 

### 5.2. K36M in Chondroblastoma

**Figure 11:**
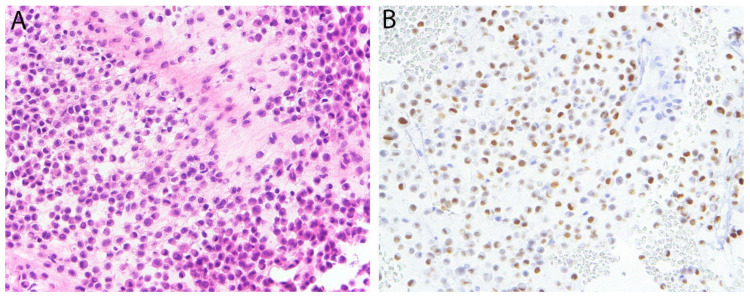
Chondroblastoma. (**A**) Sheets of chondroblastic cells are present in an eosinophilic matrix. (**B**) There is diffuse nuclear expression of K36M.

Chondroblastoma is a benign primary bone tumor also often located in the epiphyses, but which is more common in younger patients (median age 15–20 years). Histologically, it is composed of chondroblastic cells with grooved nuclei, osteoclast-type giant cells, and chondroid matrix (Figure 11). As in GCTB, histone mutations have been characterized as driver events chondroblastoma: 96% of cases harbor p.Lys36Met (p.K36M) mutations, most commonly in *H3-3B* (*H3F3B*), but rarely in the paralogous gene *H3-3A* (*H3F3A*) [80]. Immunohistochemistry using an antibody against K36M correlates very accurately with underlying mutation (in either gene) and is therefore highly sensitive and specific [83].

## 6. Markers Discovered through Gene Expression Profiling

### 6.1. DOG1 in Gastrointestinal Stromal Tumor

‘Discovered on GIST-1’ (DOG1), also known as anoctamin-1 (ANO1), is a calcium-activated chloride channel named for its expression in gastrointestinal stromal tumor (GIST) [84]. Initially discovered through gene expression profiling [85], DOG1 is expressed by around 95% of GIST [86,87]. As with KIT, DOG1 is expressed by normal interstitial cells of Cajal and care must be taken not to overinterpret their staining when entrapped in other tumor types such as leiomyomas. DOG1 is particularly useful in that it is positive in a substantial proportion (at least 50%) of KIT-negative GIST, which represent around 5% of GIST overall [84]. It should be noted that the majority of KIT-negative GIST harbor *PDGFRA* mutations [88] and this subset can also be more specifically identified with PDGFRA immunohistochemistry [89]. Both *KIT* and *PDGFRA*-mutated GIST are associated with response to imatinib therapy, with those harboring *KIT* exon 11 mutations being most sensitive [90,91].

### 6.2. NKX2.2 in Ewing Sarcoma and NKX3.1

NKX2.2 is a homeodomain transcription factor that has roles in neural tube and glial development [92]. In Ewing sarcoma, a round cell sarcoma that is most common in the diaphysis of long bones, NKX2.2 is a downstream target of the EWSR1-FLI1 fusion protein that is critical for tumor development [93]. Immunohistochemistry for NKX2.2 has been shown to be very sensitive (93%) [94,95], although it has moderate specificity; positive staining can be seen in several histologic mimics including small cell carcinoma, olfactory neuroblastoma and mesenchymal chondrosarcoma [95]. Interestingly, mesenchymal chondrosarcoma and round cell sarcomas with *EWSR1-NFATC2* fusions have been recently found to express the related protein, NKX3.1, more widely known as a marker of prostatic differentiation. Yoshida et al. first reported NKX3.1 expression in 9/11 (82%) of *EWSR1-NFATC2* sarcomas using a polyclonal antibody, consistent with transcriptomic analyses, as well as 9/9 mesenchymal chondrosarcomas [96]. After two other studies could not replicate these findings [97,98], a follow-up study by Yoshida et al. confirmed NKX3.1 expression in both tumor types using a monoclonal antibody, as well as RT-PCR and in situ hybridization [99]. The reasons for these discrepant findings remain somewhat uncertain.

### 6.3. ETV4 and WT1 in CIC-Rearranged Sarcoma

*CIC*-rearranged sarcoma is an aggressive round cell sarcoma that occurs most often in the trunk and extremities of young adults. Histologically, although it can resemble Ewing sarcoma, the cells typically have more vesicular nuclei with mild pleomorphism and prominent nucleoli, while there is also frequently a lobulated growth pattern and areas of necrosis [100]. Around 95% harbor *CIC-DUX4* fusions, with the remainder showing *CIC* fusion with alternative genes (e.g., *NUTM1*, *FOXO4*, and *LEUTX*) [101]. A common feature among these tumors is upregulation of WT1 and ETV4, which have therefore become useful diagnostic adjuncts; positivity is observed in 95% and 90% of cases, respectively, while 85% express both markers [102]. Diffuse ETV4 expression is more specific than WT1 and the specificity of co-expression is 96% [102]. Of note, since *DUX4* represents the partner gene in most cases, immunohistochemistry for DUX4 is a further diagnostic marker which has also been shown to be very reliable in a small series [103].

### 6.4. MUC4 in Low-Grade Fibromyxoid Sarcoma

**Figure 12:**
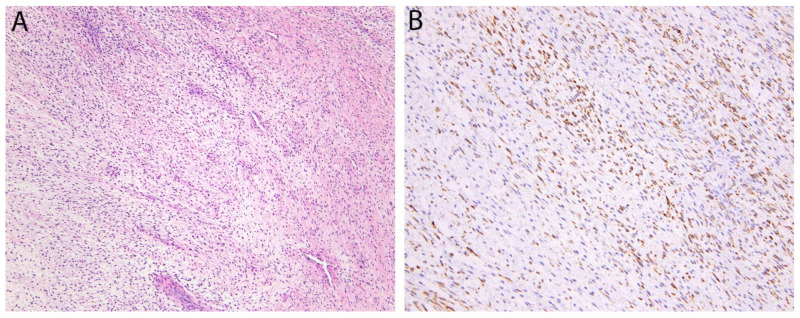
Low-grade fibromyxoid sarcoma. (**A**) The tumor consists of bland fibroblastic spindle cells with a vaguely fascicular and whorled growth pattern. Sharply alternating areas of collagenous and myxoid stroma as well as prominent branching blood vessels are also typically present. (**B**) Cytoplasmic expression of MUC4, an epithelial mucin, is seen in nearly all cases.

Mucin 4 (MUC4) is a transmembrane glycoprotein that is normally expressed by various epithelial cell types. It was therefore quite unexpected when in 2011, gene expression profiling revealed MUC4 to be highly upregulated in low-grade fibromyxoid sarcoma (LGFMS) [104]. LGFMS is a fibroblastic neoplasm that most commonly arises in deep soft tissues. Histologically, it usually has a distinctive stroma, with alternating collagenous and myxoid areas, containing bland spindle cells with a whorled or loosely fascicular growth pattern [105] (Figure 12). At the molecular level, it harbors *FUS-CREB3L2* or *FUS-CREB3L1* gene fusions. LGFMS exists on a morphologic and molecular spectrum with sclerosing epithelioid fibrosarcoma (SEF), while some tumors can exhibit hybrid features. Both LGFMS and SEF invariably show strong and diffuse cytoplasmic expression of MUC4, which is very uncommon in most histologic mimics, although positive staining can be seen in a subset of synovial sarcomas (particularly the biphasic type) [106,107]. More recently, MUC4 was also shown to be expressed in around 80% of fusion-positive alveolar rhabdomyosarcomas [108], while it was only rarely positive in other RMS subtypes. This finding again correlates with the upregulation demonstrated by gene expression profiling.

## 7. Summary

In summary, there now exists a multitude of immunohistochemical markers that can act as reliable surrogates for molecular testing in the diagnosis of sarcomas. In addition to driving improvements in everyday diagnosis, some of these markers may have significant implications for patient therapy (e.g., ALK and Pan-TRK) or suggest syndromic associations (e.g., SDHB and PRKAR1A). Given that gene fusions represent the largest category of recurrent alterations in these neoplasms, the recent introduction of fusion-specific antibodies (e.g., SS18-SSX and PAX3/7-FOXO1) is also particularly exciting, as these further demonstrate the potential for immunohistochemistry to replace molecular testing in many instances. Nevertheless, it is important to be aware of the limitations of each immunohistochemical marker, to interpret staining patterns within the clinical and histologic context, and to recognize those situations where molecular testing remains necessary.

## Figures and Tables

**Table 1:** Summary of immunohistochemical surrogates for the detection of genetic alterations in sarcomas.

Alteration	Marker	Tumor Type	Molecular Alterations
**Gene fusion**	CCNB3	BCOR-CCNB3 sarcoma	*BCOR-CCNB3* fusion
	BCOR	BCOR-CCNB3 sarcoma	*BCOR-CCNB3* fusion
		Primitive myxoid mesenchymal tumor of infancy	*BCOR* internal tandem duplications
		Clear cell sarcoma of kidney	*BCOR* internal tandem duplications
	WT1 C-terminus	Desmoplastic small round cell tumor	*EWSR1-WT1* fusion
	PAX3/7-FOXO1	Alveolar rhabdomyosarcoma	*PAX3-FOXO1* fusion (~80%) *PAX7-FOXO1* fusion (~20%)
	PAX3	Biphenotypic sinonasal sarcoma	*PAX3* fusions
	STAT6	Solitary fibrous tumor	*NAB2-STAT6* fusion
	ALK	Inflammatory myofibroblastic tumor	*ALK* fusions (~60%)
	ROS1	Inflammatory myofibroblastic tumor	*ROS1* fusions (<10%)
	Pan-Trk	Infantile fibrosarcoma	*ETV6-NTRK3* fusion
		*NTRK*-rearranged spindle cell neoplasms	*NTRK1/2/3* fusions
	SS18-SSX	Synovial sarcoma	*SS18-SSX1/2/4* fusion
	CAMTA1	Epithelioid hemangioendothelioma	*WWTR1-CAMTA1* fusion (>90%)
	TFE3	Alveolar soft part sarcoma	*ASPSCR1-TFE3* fusion
		Epithelioid hemangioendothelioma	*YAP1-TFE3* fusion (<10%)
		PEComa	*TFE3 fusions, TSC1/TSC2* inactivation
	FOSB	Pseudomyogenic hemangioendothelioma	*FOSB* fusions
		Epithelioid hemangioma	*FOSB/FOS* fusions (~50%)
	FOS	Osteoblastoma and osteoid osteoma	*FOS* rearrangements (~90%)
	DDIT3	Myxoid liposarcoma	*FUS-DDIT3* fusion
**Amplification**	MDM2/CDK4	Well-differentiated liposarcoma /Atypical lipomatous tumor	12q13-15 *(MDM2/CDK4)* amplification
		Intimal sarcoma	12q13-15 *(MDM2/CDK4)* amplification
		Low-grade central osteosarcoma and parosteal osteosarcoma	12q13-15 *(MDM2/CDK4)* amplification
**Inactivation**	SMARCB1	Epithelioid sarcoma	*SMARCB1* inactivation
		Malignant rhabdoid tumor	*SMARCB1* inactivation
		Epithelioid malignant peripheral nerve sheath tumor	*SMARCB1* inactivation
		Poorly differentiated chordoma	*SMARCB1* inactivation
	SMARCA4	Thoracic SMARCA4-deficient undifferentiated tumor	*SMARCA4* inactivation
		SMARCA4-deficient uterine sarcoma	*SMARCA4* inactivation
	SDHB	Gastrointestinal stromal tumor	*SDH* inactivation
	PRKAR1A	Malignant melanotic nerve sheath tumor	*PRKAR1A* inactivation
**Epigenetic**	H3K27me3	Malignant peripheral nerve sheath tumor	*NF1* inactivation, *SUZ12* or *EED* inactivation
**SNV**	G34W	Giant cell tumor of bone	*H3-3A* (*H3F3A*) mutations: p.Gly34Trp (G34W) ~90%
	K36M	Chondroblastoma	*H3-3B* (*H3F3B*) mutations: p.Lys36Met (K36M) ~95%
**Overexpression**	NKX2.2	Ewing sarcoma	*EWSR1-FLI1* (85%) *EWSR1-ERG* (10%) *EWSR1*-ETS gene *FUS*-ETS gene
	NKX3.1	Mesenchymal chondrosarcoma	*HEY1-NCOA2* fusion (~100%)
		EWSR1-NFATC2 and FUS-NFATC2 sarcomas	*EWSR1-NFATC2* and *FUS-NFATC2* fusions
	WT1 and ETV4	CIC-rearranged sarcomas	*CIC-DUX4 (95%)* *CIC-NUTM1, CIC-FOXO4, CIC-LEUTX*
	MUC4	Low grade fibromyxoid sarcoma sclerosing epithelioid fibrosarcoma	FUS-CREB3L2 fusion FUS-CREB3L1 fusion
	DOG1	Gastrointestinal stromal tumor	*KIT, PDGFRA, SDH, BRAF*

SNV: single nucleotide variant.

## References

[1] WHO Classification of Tumours Editorial Board (2020). World Health Organization Classification of Soft Tissue and Bone Tumours.

[2] Pierron G., Tirode F., Lucchesi C., Reynaud S., Ballet S., Cohen-Gogo S., Perrin V., Coindre J.M., Delattre O. (2012). A new subtype of bone sarcoma defined by BCOR-CCNB3 gene fusion. Nat. Genet..

[3] Kao Y.C., Owosho A.A., Sung Y.S., Zhang L., Fujisawa Y., Lee J.C., Wexler L., Argani P., Swanson D., Dickson B.C. (2018). BCOR-CCNB3 Fusion Positive Sarcomas: A Clinicopathologic and Molecular Analysis of 36 Cases with Comparison to Morphologic Spectrum and Clinical Behavior of Other Round Cell Sarcomas. Am. J. Surg. Pathol..

[4] Matsuyama A., Shiba E., Umekita Y., Nosaka K., Kamio T., Yanai H., Miyasaka C., Watanabe R., Ito I., Tamaki T. (2017). Clinicopathologic Diversity of Undifferentiated Sarcoma With BCOR-CCNB3 Fusion: Analysis of 11 Cases With a Reappraisal of the Utility of Immunohistochemistry for BCOR and CCNB3. Am. J. Surg. Pathol..

[5] Kao Y.C., Sung Y.S., Zhang L., Jungbluth A.A., Huang S.C., Argani P., Agaram N.P., Zin A., Alaggio R., Antonescu C.R. (2016). BCOR Overexpression Is a Highly Sensitive Marker in Round Cell Sarcomas with BCOR Genetic Abnormalities. Am. J. Surg. Pathol..

[6] Gerald W.L., Miller H.K., Battifora H., Miettinen M., Silva E.G., Rosai J. (1991). Intra-abdominal desmoplastic small round-cell tumor. Report of 19 cases of a distinctive type of high-grade polyphenotypic malignancy affecting young individuals. Am. J. Surg. Pathol..

[7] de Alava E., Ladanyi M., Rosai J., Gerald W.L. (1995). Detection of chimeric transcripts in desmoplastic small round cell tumor and related developmental tumors by reverse transcriptase polymerase chain reaction. A specific diagnostic assay. Am. J. Pathol..

[8] Barnoud R., Sabourin J.C., Pasquier D., Ranchère D., Bailly C., Terrier-Lacombe M.J., Pasquier B. (2000). Immunohistochemical expression of WT1 by desmoplastic small round cell tumor: A comparative study with other small round cell tumors. Am. J. Surg. Pathol..

[9] Rudzinski E.R., Anderson J.R., Chi Y.Y., Gastier-Foster J.M., Astbury C., Barr F.G., Skapek S.X., Hawkins D.S., Weigel B.J., Pappo A. (2017). Histology, fusion status, and outcome in metastatic rhabdomyosarcoma: A report from the Children’s Oncology Group. Pediatr. Blood Cancer.

[10] Buckingham M., Relaix F. (2015). PAX3 and PAX7 as upstream regulators of myogenesis. Semin. Cell Dev. Biol..

[11] Azorsa D.O., Bode P.K., Wachtel M., Cheuk A.T.C., Meltzer P.S., Vokuhl C., Camenisch U., Khov H.L., Bode B., Schäfer B.W. (2020). Immunohistochemical detection of PAX-FOXO1 fusion proteins in alveolar rhabdomyosarcoma using breakpoint specific monoclonal antibodies. Mod. Pathol..

[12] Morgenstern D.A., Gibson S., Sebire N.J., Anderson J. (2009). PAX5 expression in rhabdomyosarcoma. Am. J. Surg. Pathol..

[13] Sullivan L.M., Atkins K.A., LeGallo R.D. (2009). PAX immunoreactivity identifies alveolar rhabdomyosarcoma. Am. J. Surg. Pathol..

[14] Lewis J.T., Oliveira A.M., Nascimento A.G., Schembri-Wismayer D., Moore E.A., Olsen K.D., Garcia J.G., Lonzo M.L., Lewis J.E. (2012). Low-grade sinonasal sarcoma with neural and myogenic features: A clinicopathologic analysis of 28 cases. Am. J. Surg. Pathol..

[15] Wang X., Bledsoe K.L., Graham R.P., Asmann Y.W., Viswanatha D.S., Lewis J.E., Lewis J.T., Chou M.M., Yaszemski M.J., Jen J. (2014). Recurrent PAX3-MAML3 fusion in biphenotypic sinonasal sarcoma. Nat. Genet..

[16] Le Loarer F., Laffont S., Lesluyes T., Tirode F., Antonescu C., Baglin A.C., Delespaul L., Soubeyran I., Hostein I., Pérot G. (2019). Clinicopathologic and Molecular Features of a Series of 41 Biphenotypic Sinonasal Sarcomas Expanding Their Molecular Spectrum. Am. J. Surg. Pathol..

[17] Jo V.Y., Marino-Enriquez A., Fletcher C.D.M., Hornick J.L. (2018). Expression of PAX3 Distinguishes Biphenotypic Sinonasal Sarcoma From Histologic Mimics. Am. J. Surg. Pathol..

[18] Chmielecki J., Crago A.M., Rosenberg M., O’Connor R., Walker S.R., Ambrogio L., Auclair D., McKenna A., Heinrich M.C., Frank D.A. (2013). Whole-exome sequencing identifies a recurrent NAB2-STAT6 fusion in solitary fibrous tumors. Nat. Genet..

[19] Robinson D.R., Wu Y.M., Kalyana-Sundaram S., Cao X., Lonigro R.J., Sung Y.S., Chen C.L., Zhang L., Wang R., Su F. (2013). Identification of recurrent NAB2-STAT6 gene fusions in solitary fibrous tumor by integrative sequencing. Nat. Genet..

[20] Doyle L.A., Vivero M., Fletcher C.D., Mertens F., Hornick J.L. (2014). Nuclear expression of STAT6 distinguishes solitary fibrous tumor from histologic mimics. Mod. Pathol..

[21] Yoshida A., Tsuta K., Ohno M., Yoshida M., Narita Y., Kawai A., Asamura H., Kushima R. (2014). STAT6 immunohistochemistry is helpful in the diagnosis of solitary fibrous tumors. Am. J. Surg. Pathol..

[22] Schweizer L., Koelsche C., Sahm F., Piro R.M., Capper D., Reuss D.E., Pusch S., Habel A., Meyer J., Göck T. (2013). Meningeal hemangiopericytoma and solitary fibrous tumors carry the NAB2-STAT6 fusion and can be diagnosed by nuclear expression of STAT6 protein. Acta Neuropathol..

[23] Doyle L.A., Tao D., Marino-Enriquez A. (2014). STAT6 is amplified in a subset of dedifferentiated liposarcoma. Mod. Pathol..

[24] Griffin C.A., Hawkins A.L., Dvorak C., Henkle C., Ellingham T., Perlman E.J. (1999). Recurrent involvement of 2p23 in inflammatory myofibroblastic tumors. Cancer Res..

[25] Marino-Enriquez A., Wang W.L., Roy A., Lopez-Terrada D., Lazar A.J., Fletcher C.D., Coffin C.M., Hornick J.L. (2011). Epithelioid inflammatory myofibroblastic sarcoma: An aggressive intra-abdominal variant of inflammatory myofibroblastic tumor with nuclear membrane or perinuclear ALK. Am. J. Surg. Pathol..

[26] Doyle L.A., Marino-Enriquez A., Fletcher C.D., Hornick J.L. (2015). ALK rearrangement and overexpression in epithelioid fibrous histiocytoma. Mod. Pathol..

[27] Cohen J.N., Yeh I., Jordan R.C., Wolsky R.J., Horvai A.E., McCalmont T.H., LeBoit P.E. (2018). Cutaneous Non-Neural Granular Cell Tumors Harbor Recurrent ALK Gene Fusions. Am. J. Surg. Pathol..

[28] Acosta A.M., Demicco E.G., Dal Cin P., Hirsch M.S., Fletcher C.D.M., Jo V.Y. (2020). Pseudosarcomatous myofibroblastic proliferations of the urinary bladder are neoplasms characterized by recurrent FN1-ALK fusions. Mod. Pathol..

[29] Chang K.T.E., Tay A.Z.E., Kuick C.H., Chen H., Algar E., Taubenheim N., Campbell J., Mechinaud F., Campbell M., Super L. (2019). ALK-positive histiocytosis: An expanded clinicopathologic spectrum and frequent presence of KIF5B-ALK fusion. Mod. Pathol..

[30] Panagopoulos I., Gorunova L., Lund-Iversen M., Lobmaier I., Bjerkehagen B., Heim S. (2016). Recurrent fusion of the genes FN1 and ALK in gastrointestinal leiomyomas. Mod. Pathol..

[31] Schöffski P., Sufliarsky J., Gelderblom H., Blay J.Y., Strauss S.J., Stacchiotti S., Rutkowski P., Lindner L.H., Leahy M.G., Italiano A. (2018). Crizotinib in patients with advanced, inoperable inflammatory myofibroblastic tumours with and without anaplastic lymphoma kinase gene alterations (European Organisation for Research and Treatment of Cancer 90101 CREATE): A multicentre, single-drug, prospective, non-randomised phase 2 trial. Lancet Respir. Med..

[32] Butrynski J.E., D’Adamo D.R., Hornick J.L., Dal Cin P., Antonescu C.R., Jhanwar S.C., Ladanyi M., Capelletti M., Rodig S.J., Ramaiya N. (2010). Crizotinib in ALK-rearranged inflammatory myofibroblastic tumor. N. Engl. J. Med..

[33] Knezevich S.R., McFadden D.E., Tao W., Lim J.F., Sorensen P.H. (1998). A novel ETV6-NTRK3 gene fusion in congenital fibrosarcoma. Nat. Genet..

[34] Rudzinski E.R., Lockwood C.M., Stohr B.A., Vargas S.O., Sheridan R., Black J.O., Rajaram V., Laetsch T.W., Davis J.L. (2018). Pan-Trk Immunohistochemistry Identifies NTRK Rearrangements in Pediatric Mesenchymal Tumors. Am. J. Surg. Pathol..

[35] Hung Y.P., Fletcher C.D.M., Hornick J.L. (2018). Evaluation of pan-TRK immunohistochemistry in infantile fibrosarcoma, lipofibromatosis-like neural tumour and histological mimics. Histopathology.

[36] Bielack S.S., Cox M.C., Nathrath M., Apel K., Blattmann C., Holl T., Jenewein R., Klenk U., Klothaki P., Müller-Abt P. (2019). Rapid, complete and sustained tumour response to the TRK inhibitor larotrectinib in an infant with recurrent, chemotherapy-refractory infantile fibrosarcoma carrying the characteristic ETV6-NTRK3 gene fusion. Ann. Oncol. Off. J. Eur. Soc. Med Oncol..

[37] Laetsch T.W., DuBois S.G., Mascarenhas L., Turpin B., Federman N., Albert C.M., Nagasubramanian R., Davis J.L., Rudzinski E., Feraco A.M. (2018). Larotrectinib for paediatric solid tumours harbouring NTRK gene fusions: Phase 1 results from a multicentre, open-label, phase 1/2 study. Lancet Oncol..

[38] Perret R., Velasco V., Le Guellec S., Coindre J.M., Le Loarer F. (2020). The SS18-SSX Antibody Has Perfect Specificity for the SS18-SSX Fusion Protein: A Validation Study of 609 Neoplasms Including 2 Unclassified Tumors With SS18-Non-SSX Fusions. Am. J. Surg. Pathol..

[39] Zaborowski M., Vargas A.C., Pulvers J., Clarkson A., de Guzman D., Sioson L., Maclean F., Chou A., Gill A.J. (2020). When used together SS18-SSX fusion-specific and SSX C-terminus immunohistochemistry are highly specific and sensitive for the diagnosis of synovial sarcoma and can replace FISH or molecular testing in most cases. Histopathology.

[40] Weiss S.W., Enzinger F.M. (1982). Epithelioid hemangioendothelioma: A vascular tumor often mistaken for a carcinoma. Cancer.

[41] Errani C., Zhang L., Sung Y.S., Hajdu M., Singer S., Maki R.G., Healey J.H., Antonescu C.R. (2011). A novel WWTR1-CAMTA1 gene fusion is a consistent abnormality in epithelioid hemangioendothelioma of different anatomic sites. Genes Chromos. Cancer.

[42] Doyle L.A., Fletcher C.D., Hornick J.L. (2016). Nuclear Expression of CAMTA1 Distinguishes Epithelioid Hemangioendothelioma From Histologic Mimics. Am. J. Surg. Pathol..

[43] Antonescu C.R., Le Loarer F., Mosquera J.M., Sboner A., Zhang L., Chen C.L., Chen H.W., Pathan N., Krausz T., Dickson B.C. (2013). Novel YAP1-TFE3 fusion defines a distinct subset of epithelioid hemangioendothelioma. Genes Chromos. Cancer.

[44] Ladanyi M., Lui M.Y., Antonescu C.R., Krause-Boehm A., Meindl A., Argani P., Healey J.H., Ueda T., Yoshikawa H., Meloni-Ehrig A. (2001). The der(17)t(X;17)(p11;q25) of human alveolar soft part sarcoma fuses the TFE3 transcription factor gene to ASPL, a novel gene at 17q25. Oncogene.

[45] Argani P., Aulmann S., Illei P.B., Netto G.J., Ro J., Cho H.Y., Dogan S., Ladanyi M., Martignoni G., Goldblum J.R. (2010). A distinctive subset of PEComas harbors TFE3 gene fusions. Am. J. Surg. Pathol..

[46] Mirra J.M., Kessler S., Bhuta S., Eckardt J. (1992). The fibroma-like variant of epithelioid sarcoma. A fibrohistiocytic/myoid cell lesion often confused with benign and malignant spindle cell tumors. Cancer.

[47] Hornick J.L., Fletcher C.D. (2011). Pseudomyogenic hemangioendothelioma: A distinctive, often multicentric tumor with indolent behavior. Am. J. Surg. Pathol..

[48] Walther C., Tayebwa J., Lilljebjorn H., Magnusson L., Nilsson J., von Steyern F.V., Ora I., Domanski H.A., Fioretos T., Nord K.H. (2014). A novel SERPINE1-FOSB fusion gene results in transcriptional up-regulation of FOSB in pseudomyogenic haemangioendothelioma. J. Pathol..

[49] Agaram N.P., Zhang L., Cotzia P., Antonescu C.R. (2018). Expanding the Spectrum of Genetic Alterations in Pseudomyogenic Hemangioendothelioma With Recurrent Novel ACTB-FOSB Gene Fusions. Am. J. Surg. Pathol..

[50] Panagopoulos I., Lobmaier I., Gorunova L., Heim S. (2019). Fusion of the Genes WWTR1 and FOSB in Pseudomyogenic Hemangioendothelioma. Cancer Genom. Proteom..

[51] Bridge J.A., Sumegi J., Royce T., Baker M., Linos K. (2021). A novel CLTC-FOSB gene fusion in pseudomyogenic hemangioendothelioma of bone. Genes Chromos. Cancer.

[52] Hung Y.P., Fletcher C.D., Hornick J.L. (2017). FOSB is a Useful Diagnostic Marker for Pseudomyogenic Hemangioendothelioma. Am. J. Surg. Pathol..

[53] Huang S.C., Zhang L., Sung Y.S., Chen C.L., Krausz T., Dickson B.C., Kao Y.C., Agaram N.P., Fletcher C.D., Antonescu C.R. (2015). Frequent FOS Gene Rearrangements in Epithelioid Hemangioma: A Molecular Study of 58 Cases With Morphologic Reappraisal. Am. J. Surg. Pathol..

[54] Fittall M.W., Mifsud W., Pillay N., Ye H., Strobl A.C., Verfaillie A., Demeulemeester J., Zhang L., Berisha F., Tarabichi M. (2018). Recurrent rearrangements of FOS and FOSB define osteoblastoma. Nat. Commun..

[55] Amary F., Markert E., Berisha F., Ye H., Gerrand C., Cool P., Tirabosco R., Lindsay D., Pillay N., O’Donnell P. (2019). FOS Expression in Osteoid Osteoma and Osteoblastoma: A Valuable Ancillary Diagnostic Tool. Am. J. Surg. Pathol..

[56] Scapa J.V., Cloutier J.M., Raghavan S.S., Peters-Schulze G., Varma S., Charville G.W. (2020). DDIT3 Immunohistochemistry Is a Useful Tool for the Diagnosis of Myxoid Liposarcoma. Am. J. Surg. Pathol..

[57] Mittal P., Roberts C.W.M. (2020). The SWI/SNF complex in cancer—Biology, biomarkers and therapy. Nat. Rev. Clin. Oncol..

[58] Versteege I., Sévenet N., Lange J., Rousseau-Merck M.F., Ambros P., Handgretinger R., Aurias A., Delattre O. (1998). Truncating mutations of hSNF5/INI1 in aggressive paediatric cancer. Nature.

[59] Hornick J.L., Dal Cin P., Fletcher C.D. (2009). Loss of INI1 expression is characteristic of both conventional and proximal-type epithelioid sarcoma. Am. J. Surg. Pathol..

[60] Schaefer I.M., Dong F., Garcia E.P., Fletcher C.D.M., Jo V.Y. (2019). Recurrent SMARCB1 Inactivation in Epithelioid Malignant Peripheral Nerve Sheath Tumors. Am. J. Surg. Pathol..

[61] Hasselblatt M., Thomas C., Hovestadt V., Schrimpf D., Johann P., Bens S., Oyen F., Peetz-Dienhart S., Crede Y., Wefers A. (2016). Poorly differentiated chordoma with SMARCB1/INI1 loss: A distinct molecular entity with dismal prognosis. Acta Neuropathol..

[62] Kohashi K., Oda Y., Yamamoto H., Tamiya S., Oshiro Y., Izumi T., Taguchi T., Tsuneyoshi M. (2008). SMARCB1/INI1 protein expression in round cell soft tissue sarcomas associated with chromosomal translocations involving EWS: A special reference to SMARCB1/INI1 negative variant extraskeletal myxoid chondrosarcoma. Am. J. Surg. Pathol..

[63] Gleason B.C., Fletcher C.D. (2007). Myoepithelial carcinoma of soft tissue in children: An aggressive neoplasm analyzed in a series of 29 cases. Am. J. Surg. Pathol..

[64] Kolin D.L., Dong F., Baltay M., Lindeman N., MacConaill L., Nucci M.R., Crum C.P., Howitt B.E. (2018). SMARCA4-deficient undifferentiated uterine sarcoma (malignant rhabdoid tumor of the uterus): A clinicopathologic entity distinct from undifferentiated carcinoma. Mod. Pathol..

[65] Rooper L.M., Uddin N., Gagan J., Brosens L.A.A., Magliocca K.R., Edgar M.A., Thompson L.D.R., Agaimy A., Bishop J.A. (2020). Recurrent Loss of SMARCA4 in Sinonasal Teratocarcinosarcoma. Am. J. Surg. Pathol..

[66] Kohashi K., Yamamoto H., Yamada Y., Kinoshita I., Taguchi T., Iwamoto Y., Oda Y. (2018). SWI/SNF Chromatin-remodeling Complex Status in SMARCB1/INI1-preserved Epithelioid Sarcoma. Am. J. Surg. Pathol..

[67] Rekhtman N., Montecalvo J., Chang J.C., Alex D., Ptashkin R.N., Ai N., Sauter J.L., Kezlarian B., Jungbluth A., Desmeules P. (2020). SMARCA4-Deficient Thoracic Sarcomatoid Tumors Represent Primarily Smoking-Related Undifferentiated Carcinomas Rather Than Primary Thoracic Sarcomas. J. Thorac. Oncol..

[68] Wagner A.J., Remillard S.P., Zhang Y.X., Doyle L.A., George S., Hornick J.L. (2013). Loss of expression of SDHA predicts SDHA mutations in gastrointestinal stromal tumors. Mod. Pathol..

[69] Torres-Mora J., Dry S., Li X., Binder S., Amin M., Folpe A.L. (2014). Malignant melanotic schwannian tumor: A clinicopathologic, immunohistochemical, and gene expression profiling study of 40 cases, with a proposal for the reclassification of “melanotic schwannoma”. Am. J. Surg. Pathol..

[70] Wang L., Zehir A., Sadowska J., Zhou N., Rosenblum M., Busam K., Agaram N., Travis W., Arcila M., Dogan S. (2015). Consistent copy number changes and recurrent PRKAR1A mutations distinguish Melanotic Schwannomas from Melanomas: SNP-array and next generation sequencing analysis. Genes Chromos. Cancer.

[71] Maleszewski J.J., Larsen B.T., Kip N.S., Castonguay M.C., Edwards W.D., Carney J.A., Kipp B.R. (2014). PRKAR1A in the development of cardiac myxoma: A study of 110 cases including isolated and syndromic tumors. Am. J. Surg. Pathol..

[72] Lee W., Teckie S., Wiesner T., Ran L., Prieto Granada C.N., Lin M., Zhu S., Cao Z., Liang Y., Sboner A. (2014). PRC2 is recurrently inactivated through EED or SUZ12 loss in malignant peripheral nerve sheath tumors. Nat. Genet..

[73] Schaefer I.M., Fletcher C.D., Hornick J.L. (2016). Loss of H3K27 trimethylation distinguishes malignant peripheral nerve sheath tumors from histologic mimics. Mod. Pathol..

[74] Cleven A.H., Sannaa G.A., Briaire-de Bruijn I., Ingram D.R., van de Rijn M., Rubin B.P., de Vries M.W., Watson K.L., Torres K.E., Wang W.L. (2016). Loss of H3K27 tri-methylation is a diagnostic marker for malignant peripheral nerve sheath tumors and an indicator for an inferior survival. Mod. Pathol..

[75] Prieto-Granada C.N., Wiesner T., Messina J.L., Jungbluth A.A., Chi P., Antonescu C.R. (2016). Loss of H3K27me3 Expression Is a Highly Sensitive Marker for Sporadic and Radiation-induced MPNST. Am. J. Surg. Pathol..

[76] Lyskjaer I., Lindsay D., Tirabosco R., Steele C.D., Lombard P., Strobl A.C., Rocha A.M., Davies C., Ye H., Bekers E. (2020). H3K27me3 expression and methylation status in histological variants of malignant peripheral nerve sheath tumours. J. Pathol..

[77] Panse G., Mito J.K., Ingram D.R., Wani K., Khan S., Lazar A.J., Doyle L.A., Wang W.L. (2020). Radiation-associated sarcomas other than malignant peripheral nerve sheath tumours demonstrate loss of histone H3K27 trimethylation(†). Histopathology.

[78] Le Guellec S., Macagno N., Velasco V., Lamant L., Lae M., Filleron T., Malissen N., Cassagnau E., Terrier P., Chevreau C. (2017). Loss of H3K27 trimethylation is not suitable for distinguishing malignant peripheral nerve sheath tumor from melanoma: A study of 387 cases including mimicking lesions. Mod. Pathol..

[79] Marchione D.M., Lisby A., Viaene A.N., Santi M., Nasrallah M., Wang L.P., Williams E.A., Larque A.B., Chebib I., Garcia B.A. (2019). Histone H3K27 dimethyl loss is highly specific for malignant peripheral nerve sheath tumor and distinguishes true PRC2 loss from isolated H3K27 trimethyl loss. Mod. Pathol..

[80] Behjati S., Tarpey P.S., Presneau N., Scheipl S., Pillay N., Van Loo P., Wedge D.C., Cooke S.L., Gundem G., Davies H. (2013). Distinct H3F3A and H3F3B driver mutations define chondroblastoma and giant cell tumor of bone. Nat. Genet..

[81] Presneau N., Baumhoer D., Behjati S., Pillay N., Tarpey P., Campbell P.J., Jundt G., Hamoudi R., Wedge D.C., Loo P.V. (2015). Diagnostic value of H3F3A mutations in giant cell tumour of bone compared to osteoclast-rich mimics. J. Pathol. Clin. Res..

[82] Amary F., Berisha F., Ye H., Gupta M., Gutteridge A., Baumhoer D., Gibbons R., Tirabosco R., O’Donnell P., Flanagan A.M. (2017). H3F3A (Histone 3.3) G34W Immunohistochemistry: A Reliable Marker Defining Benign and Malignant Giant Cell Tumor of Bone. Am. J. Surg. Pathol..

[83] Amary M.F., Berisha F., Mozela R., Gibbons R., Guttridge A., O’Donnell P., Baumhoer D., Tirabosco R., Flanagan A.M. (2016). The H3F3 K36M mutant antibody is a sensitive and specific marker for the diagnosis of chondroblastoma. Histopathology.

[84] Kang G.H., Srivastava A., Kim Y.E., Park H.J., Park C.K., Sohn T.S., Kim S., Kang D.Y., Kim K.M. (2011). DOG1 and PKC-θ are useful in the diagnosis of KIT-negative gastrointestinal stromal tumors. Mod. Pathol..

[85] Nielsen T.O., West R.B., Linn S.C., Alter O., Knowling M.A., O’Connell J.X., Zhu S., Fero M., Sherlock G., Pollack J.R. (2002). Molecular characterisation of soft tissue tumours: A gene expression study. Lancet.

[86] West R.B., Corless C.L., Chen X., Rubin B.P., Subramanian S., Montgomery K., Zhu S., Ball C.A., Nielsen T.O., Patel R. (2004). The novel marker, DOG1, is expressed ubiquitously in gastrointestinal stromal tumors irrespective of KIT or PDGFRA mutation status. Am. J. Pathol..

[87] Miettinen M., Wang Z.F., Lasota J. (2009). DOG1 antibody in the differential diagnosis of gastrointestinal stromal tumors: A study of 1840 cases. Am. J. Surg. Pathol..

[88] Medeiros F., Corless C.L., Duensing A., Hornick J.L., Oliveira A.M., Heinrich M.C., Fletcher J.A., Fletcher C.D. (2004). KIT-negative gastrointestinal stromal tumors: Proof of concept and therapeutic implications. Am. J. Surg. Pathol..

[89] Agaimy A., Otto C., Braun A., Geddert H., Schaefer I.M., Haller F. (2013). Value of epithelioid morphology and PDGFRA immunostaining pattern for prediction of PDGFRA mutated genotype in gastrointestinal stromal tumors (GISTs). Int. J. Clin. Exp. Pathol..

[90] DeMatteo R.P., Ballman K.V., Antonescu C.R., Corless C., Kolesnikova V., von Mehren M., McCarter M.D., Norton J., Maki R.G., Pisters P.W. (2013). Long-term results of adjuvant imatinib mesylate in localized, high-risk, primary gastrointestinal stromal tumor: ACOSOG Z9000 (Alliance) intergroup phase 2 trial. Ann. Surg..

[91] Dematteo R.P., Ballman K.V., Antonescu C.R., Maki R.G., Pisters P.W., Demetri G.D., Blackstein M.E., Blanke C.D., von Mehren M., Brennan M.F. (2009). Adjuvant imatinib mesylate after resection of localised, primary gastrointestinal stromal tumour: A randomised, double-blind, placebo-controlled trial. Lancet.

[92] Briscoe J., Sussel L., Serup P., Hartigan-O’Connor D., Jessell T.M., Rubenstein J.L., Ericson J. (1999). Homeobox gene Nkx2.2 and specification of neuronal identity by graded Sonic hedgehog signalling. Nature.

[93] Smith R., Owen L.A., Trem D.J., Wong J.S., Whangbo J.S., Golub T.R., Lessnick S.L. (2006). Expression profiling of EWS/FLI identifies NKX2.2 as a critical target gene in Ewing’s sarcoma. Cancer Cell.

[94] Yoshida A., Sekine S., Tsuta K., Fukayama M., Furuta K., Tsuda H. (2012). NKX2.2 is a useful immunohistochemical marker for Ewing sarcoma. Am. J. Surg. Pathol..

[95] Hung Y.P., Fletcher C.D., Hornick J.L. (2016). Evaluation of NKX2-2 expression in round cell sarcomas and other tumors with EWSR1 rearrangement: Imperfect specificity for Ewing sarcoma. Mod. Pathol..

[96] Yoshida K.I., Machado I., Motoi T., Parafioriti A., Lacambra M., Ichikawa H., Kawai A., Antonescu C.R., Yoshida A. (2020). NKX3-1 Is a Useful Immunohistochemical Marker of EWSR1-NFATC2 Sarcoma and Mesenchymal Chondrosarcoma. Am. J. Surg. Pathol..

[97] Perret R., Escuriol J., Velasco V., Mayeur L., Soubeyran I., Delfour C., Aubert S., Polivka M., Karanian M., Meurgey A. (2020). NFATc2-rearranged sarcomas: Clinicopathologic, molecular, and cytogenetic study of 7 cases with evidence of AGGRECAN as a novel diagnostic marker. Mod. Pathol..

[98] Chen W., Hornick J.L., Fletcher C.D.M. (2021). NKX3.1 immunoreactivity is not identified in mesenchymal chondrosarcoma: A 25-case cohort study. Histopathology.

[99] Yoshida A., Hashimoto T., Ryo E., Yoshida K.I., Motoi T., Yatabe Y., Mori T. (2021). Confirmation of NKX3-1 Expression in EWSR1-NFATC2 Sarcoma and Mesenchymal Chondrosarcoma Using Monoclonal Antibody Immunohistochemistry, RT-PCR, and RNA In Situ Hybridization. Am. J. Surg. Pathol..

[100] Antonescu C.R., Owosho A.A., Zhang L., Chen S., Deniz K., Huryn J.M., Kao Y.C., Huang S.C., Singer S., Tap W. (2017). Sarcomas With CIC-rearrangements Are a Distinct Pathologic Entity With Aggressive Outcome: A Clinicopathologic and Molecular Study of 115 Cases. Am. J. Surg. Pathol..

[101] Italiano A., Sung Y.S., Zhang L., Singer S., Maki R.G., Coindre J.M., Antonescu C.R. (2012). High prevalence of CIC fusion with double-homeobox (DUX4) transcription factors in EWSR1-negative undifferentiated small blue round cell sarcomas. Genes Chromos. Cancer.

[102] Hung Y.P., Fletcher C.D., Hornick J.L. (2016). Evaluation of ETV4 and WT1 expression in CIC-rearranged sarcomas and histologic mimics. Mod. Pathol..

[103] Siegele B., Roberts J., Black J.O., Rudzinski E., Vargas S.O., Galambos C. (2017). DUX4 Immunohistochemistry Is a Highly Sensitive and Specific Marker for CIC-DUX4 Fusion-positive Round Cell Tumor. Am. J. Surg. Pathol..

[104] Möller E., Hornick J.L., Magnusson L., Veerla S., Domanski H.A., Mertens F. (2011). FUS-CREB3L2/L1-positive sarcomas show a specific gene expression profile with upregulation of CD24 and FOXL1. Clin. Cancer Res..

[105] Evans H.L. (1987). Low-grade fibromyxoid sarcoma. A report of two metastasizing neoplasms having a deceptively benign appearance. Am. J. Clin. Pathol..

[106] Doyle L.A., Wang W.L., Dal Cin P., Lopez-Terrada D., Mertens F., Lazar A.J., Fletcher C.D., Hornick J.L. (2012). MUC4 is a sensitive and extremely useful marker for sclerosing epithelioid fibrosarcoma: Association with FUS gene rearrangement. Am. J. Surg. Pathol..

[107] Doyle L.A., Möller E., Dal Cin P., Fletcher C.D., Mertens F., Hornick J.L. (2011). MUC4 is a highly sensitive and specific marker for low-grade fibromyxoid sarcoma. Am. J. Surg. Pathol..

[108] Forgó E., Hornick J.L., Charville G.W. (2020). MUC4 is expressed in alveolar rhabdomyosarcoma. Histopathology.

